# Improving risk equalization with constrained regression

**DOI:** 10.1007/s10198-016-0859-1

**Published:** 2016-12-10

**Authors:** Richard C. van Kleef, Thomas G. McGuire, René C. J. A. van Vliet, Wynand P. P. M. van de Ven

**Affiliations:** 1 Institute of Health Policy and Management, Erasmus University Rotterdam, P.O. Box 1738, 3000 DR Rotterdam, The Netherlands; 2 Department of Health Care Policy, Harvard Medical School, 180 Longwood Avenue, Boston, MA 02115 USA; 3 National Bureau of Economic Research, 1050 Massachusetts Avenue, Cambridge, MA 02138 USA

**Keywords:** Health insurance, Risk equalization, Capitation, Risk selection, Constrained regression, I11, I13, G22

## Abstract

State-of-the-art risk equalization models undercompensate some risk groups and overcompensate others, leaving systematic incentives for risk selection. A natural approach to reducing the under- or overcompensation for a particular group is enriching the risk equalization model with risk adjustor variables that indicate membership in that group. For some groups, however, appropriate risk adjustor variables may not (yet) be available. For these situations, this paper proposes an alternative approach to reducing under- or overcompensation: constraining the estimated coefficients of the risk equalization model such that the under- or overcompensation for a group of interest equals a fixed amount. We show that, compared to ordinary least-squares, constrained regressions can reduce under/overcompensation for some groups but increase under/overcompensation for others. In order to quantify this trade-off two fundamental questions need to be answered: “Which groups are relevant in terms of risk selection actions?” and “What is the relative importance of under- and overcompensation for these groups?” By making assumptions on these aspects we empirically evaluate a particular set of constraints using individual-level data from the Netherlands (*N* = 16.5 million). We find that the benefits of introducing constraints in terms of reduced under/overcompensations for some groups can be worth the costs in terms of increased under/overcompensations for others. Constrained regressions add a tool for developing risk equalization models that can improve the overall economic performance of health plan payment schemes.

## Introduction

Several countries have adopted elements of Alain Enthoven’s model of regulated health plan competition [10] which combines affordability of health plans with incentives for cost containment and quality improvement.^1^ A crucial element of Enthoven’s model is the adjustment of health plan payments to predictable variation in medical spending, also referred to as risk equalization (RE). In the absence of premium regulation, RE mitigates incentives for health plans to risk rate their premiums and thereby improves affordability of health plans for the sick. In the presence of premium regulation — as is common in regulated health plan markets — RE mitigates incentives for risk selection and thereby improves incentives for health plans to accept and serve the sick as well as the healthy [20].^2^

Recent research has shown that even state-of-the-art RE models — such as those used under the Affordable Care Act in the United States or those used under the Health Insurance Act in the Netherlands — systematically undercompensate groups of consumers in relatively poor health and overcompensate the complementary groups of consumers in relatively good health [16, 30], exposing health plans and consumers to incentives for risk selection. As described by van de Ven and Ellis [28], risk selection threatens the performance of (regulated) health plan markets since it may reduce (1) the quality of care (because plans may have a disincentive to meet the preferences of the sick), (2) the efficiency of care (because risk selection may be a more cost-effective strategy for plans to reduce medical spending than improving the efficiency of care), (3) the efficient sorting of consumers among plans (when market segmentation by risk elevates premiums for particular plans), and (4) the affordability of health plans to the sick (when the same market segmentation causes the sick to face higher premiums). To contend with these potential problems, researchers and policy makers work to improve the properties of health plan payment schemes.^3^ In general, three strategies can be applied to reduce incentives for risk selection in regulated health plan markets: improving RE, increasing risk sharing (e.g., via mandatory reinsurance or risk corridors) and relaxing premium-rate restrictions [28]. This paper focuses on the first strategy.

The conventional approach to reducing under- or overcompensation for specific groups is enriching the RE model with new/better risk adjustor variables that indicate membership in these groups. If a group of interest (e.g., persons with congestive heart failure) is explicitly recognized by a risk adjustor variable (e.g., via a diagnostic cost group for “congestive heart failure”), ordinary least squares (OLS, the common estimation method for RE models) ensures that the payment for this group will equal the average medical spending of this group.^4^ For some groups, however, appropriate risk adjustor variables may not (yet) be available. In this paper we study two concrete examples in the setting of the Dutch national health insurance: “users of home care in the previous year” and “users of physiotherapy in the previous year”. These groups are known to be substantially undercompensated by the Dutch RE model. So far, however, the Dutch government has not found *appropriate* risk adjustor variables to indicate membership in these particular groups. One option considered by the Dutch government is using the indicators “yes/no use of home care in the previous year” and “yes/no use of physiotherapy in the previous year” as risk adjustor variables. The Dutch Minister of Health, however, has acknowledged that these indicators are *inappropriate* for inclusion in the RE model since they would introduce substantial incentives to overuse these services [37].^5^ Though, as long as the undercompensation of the groups in question continues to persist, health plans are confronted with incentives for risk selection, e.g., by skimping on the quality of home care and physiotherapy.

Van Kleef et al. [34] have proposed reducing the under- or overcompensation for a group in case *appropriate* risk adjustor variables to identify this group are not available by introducing under/overpayments for existing risk adjustor variables that are correlated with membership in this group. For example, if “yes/no use of home care in the previous year” is positively correlated with the risk adjustor variable “yes/no inclusion in any diagnostic cost group (DCG)”, the undercompensation for the home care group can be reduced by increasing the risk equalization payment for people with a DCG. Note that — given the zero sum principle on which RE systems in practice are based — the increase in payments for people with a DCG comes with a decrease of payments for people without a DCG and thus a reduction of the overcompensation for non-users of home care in the previous year. Though this strategy is intuitively appealing, van Kleef et al. did not provide an analytical solution for calculating the changes in weights for existing risk adjustor variables. In this paper we propose and illustrate an analytical approach to solve this problem that can be easily implemented in practice: constrained least-squares regression.

In practice, RE models are estimated by OLS regression using a series of indicators as risk adjustor variables. As described by van de Ven and Ellis [28], not all indicators are appropriate for serving as a risk adjustor variable. Examples of inappropriate indicators may include information directly based on costs and utilization which would introduce incentives for overuse and gaming (i.e., discretionary coding by plans or providers seeking to enhance revenues). Recent research, for instance, finds substantial “upcoding” in health plans paid by capitation in the US Medicare program [11]. Another example is health survey information, which may identify a group of interest and be a highly informative predictor of medical spending, but too expensive to collect on a regular basis for everyone in a risk pool. Conventional RE ignores such inappropriate indicators during model estimation. Constrained regression allows for *indirect* use of such indicators by constraining the estimated coefficients of the RE model to reduce under- or overcompensation of the groups identified by these indicators. This constrained regression can improve compensation for these groups by exploiting the empirical correlation between omitted and included indicators. At the same time, however, constraints will introduce under- or overcompensation for included groups (compared to OLS). In Section “Theory and concepts” of this paper we will argue that in order to quantify this trade-off two questions need to be answered: “Which groups are relevant in terms of risk selection actions?” and “What is the relative importance of under- and overcompensation for these groups?” By making assumptions on these aspects, we will show empirically that the gains from a well-chosen constraint in terms of improved payment fit for omitted indicators can be worth the costs in terms of reduced payment fit for included indicators.

Our empirical application is the national basic health insurance for curative care in the Netherlands, a well-established example of a regulated individual health plan market based on principles of regulated competition [27]. In spite of a sophisticated RE model, policy researchers have identified groups that are systematically under- or overcompensated [30]. The two groups we study in this paper are known to be undercompensated by about 1200 euro (users of home care in the previous year) and 900 euro (users of physiotherapy in the previous year) per person per year. So far, the Dutch government has not yet found appropriate risk adjustor variables to improve compensation for these groups.

The paper is structured as follows. Section “Theory and concepts” discusses the method of constrained regression and develops measures for quantifying the trade-off between improved payment fit for omitted indicators and reduced payment fit for included indicators when using constrained regressions in the context of RE. In Sections “Data and empirical methods” and “Results” we apply our approach to the Dutch RE model of 2015 using data on medical spending and characteristics of nearly all individuals with basic health insurance in the Netherlands (*N* = 16.5 million). We explore using constrained regressions to address the undercompensation of the users of home care or physiotherapy in the previous year. We apply our measures from Section “Theory and concepts” to show that, generally, some reduction in undercompensation for indicators omitted from the RE model can be worth the increase in under- or overcompensation for indicators included in the model. Section “Discussion” discusses our main findings and their implications.

## Theory and concepts

### Constrained regression

Least squares regression methods choose values for a set of parameters, the estimated coefficients, to minimize the residual sum of squared differences between the actual and fitted values from the regression. A researcher may place constraints on the choice of the coefficients in this minimization for various reasons. One common reason for imposing a constraint is to test a hypothesis about a set of coefficients. For example, to test the hypothesis that earned and unearned income has the same effect on household consumption, a constraint can impose the restriction that the coefficients on these two types of income are the same. The researcher can compare the model fit with and without the constraint using an F-statistic to test whether the reduction in explained variance is statistically significant; if it is, the hypothesis of constant returns is rejected.

Our motivation for introducing a constraint is different, and is akin to methods of constrained optimization. Health plan payment schemes have multiple objectives subject to trade-offs. For example, in the design of a public health insurance program, one objective may be to reduce financial risk of the population while another objective may be to reduce public expenditures, with a trade-off between the two. The locus of efficient policies can be found by maximizing one objective subject to a given level of attainment of the other, by, for example, maximizing financial protection for the population for a given level of public expenditures.^6^ By conducting this maximization for different levels of public expenditure, the researcher can characterize the trade-off between spending more public money and reducing financial risk of the population.

Introducing constraints into a RE model serves a similar purpose. Constrained least squares regression addresses selection incentives regarding *included* and *omitted* indicators simultaneously by pursuing the “usual” objective of a RE model — minimizing squared deviations at the individual level for the *included* indicators — subject to a maximum value of under- or overcompensation for the *omitted* indicators. By varying the maximum value of this second objective, researchers can trace out the trade-offs between fit on the included indicators and over- or undercompensation on the omitted ones.^7^

Constrained regressions have been studied previously in the context of RE. Glazer et al. [13] proposed using constrained regression to address selection problems, where the constraints were derived from first-order conditions for plan profit maximization, with one constraint for each service provided by the plan. RE weights were best fitting given a set of linear constraints that guaranteed a balanced set of incentives for plans to fund all services. This theoretical approach has never been implemented empirically, probably due to the complexity of specifying the constraints. In addition, there was no obvious way to “tighten” or “loosen” the constraints as there is here with the magnitude of undercompensation being the target of the constraint. McGuire et al. [18] and Eijkenaar et al. [8] have used constrained regressions in the context of RE, though for purposes other than addressing selection incentives.

This paper focuses on situations where compensation for omitted variables is *desired*. This starting point is distinct from that of Schokkaert and van de Voorde [25] who study situations where compensation for omitted variables is *not desired*. They argue that when omitted indicators for which compensation is not desired (which they refer to as *R*-variables)^8^ are correlated with indicators included in the RE model for which compensation is desired (*C*-variables), conventional RE leads to biased estimates of coefficients for *C*-variables since these will (partly) pick up the variation in spending due to the omitted *R*-variables. In Schokkaert and van de Voorde’s terminology our paper exclusively focuses on *C*-variables.

### Evaluating incentives for selection in RE models

A major purpose of RE models is to mitigate incentives for plans to over- or underserve groups among the population. Incentives to underserve enrollees with a mental illness, for example, are created if the payments a plan receives for members of this group fall below the costs they bring to the plan. A RE model that recognizes and pays more for persons with some mental illness diagnoses can reduce the gap between average costs and average payments. However, if the RE model recognizes some but not all mental conditions, a plan might seek to deter persons with mental illness from joining by not contracting with first-best providers of mental health services—an example of inefficiency created by selection incentives. Incentives for a plan to “distort” its benefits away from the efficient mix to attract/deter have been studied theoretically since Rothschild and Stiglitz [24],^9^ and empirically since the beginning of the use of RE in public insurance programs [21]. In the US context, empirical evidence confirms that plans respond to this type of incentive in service provision.^10^ In the Netherlands, several health plans have reported publicly that the imperfect RE discourages them from improving the quality of care for groups that are systematically undercompensated [29].

Papers and reports concerned with incentives for selection first define the group or groups of concern and then compare average payment for members of the group to average medical spending by simulation methods. Evaluations of payment systems in Medicare and in the marketplaces in the US commonly employ “predictive ratios”, a ratio with simulated RE payments for the group in the numerator and medical spending in the denominator. “Underpayment” is indicated if the predictive ratio is less than 1.0. Evaluating the RE model proposed for the marketplaces, Kautter et al. [14] created subgroups of individuals by predicted spending and computed predictive ratios for these subgroups.^11^ In an evaluation of the CMS-HCC model used in Medicare, Pope et al. [22] report predictive ratios for subgroups defined by disease, numbers of prior hospitalizations, demographic characteristics, and other factors.^12^

Other papers calculate the difference between RE payments and spending to assess selection incentives, with the difference being referred to as “undercompensation” if payments are less than spending and “overcompensation” in the opposite case. Van Kleef et al. [30] merged survey information with health claims for a subset of people in the Netherlands to calculate undercompensation for various groups of people, including those with low physical and mental self-rated health statuses and those reporting chronic conditions. In the current paper we track over- and undercompensation, both for defining constraints and as a basis for our evaluation metrics.

In empirical research, both forms of measures, predictive ratios and monetary differences, are primarily applied to groups for whom an indicator is *not* included in the RE model. The reason is that under the ordinary least squares approach, RE models eliminate under- and overcompensation for groups with indicators *included* in the RE model (see footnote 4). Under a *constrained* least squares approach, however, over- and undercompensation can appear for the latter as well.

Missing from the literature is an accepted method for aggregating group-level measures of under- and overcompensation to the entire population, or, in other words, there is no accepted summary measure for comparing the comprehensive performance of alternative RE models affecting multiple groups simultaneously. While we can agree that reducing undercompensation for a group of interest is an improvement for that particular group, what if a RE alternative decreases undercompensation for one group but increases it for another? Which RE model is preferred? These questions are directly relevant for this paper since the type of constraints applied here are expected to improve compensation for omitted groups but will generally worsen it for included groups. A weighted sum of under- and overcompensations for all groups of concern (both omitted and included groups) is a natural basis for construction of a summary measure with the weight being the share of the population in the group of interest. In the next section, we propose a family of such measures that we apply later to empirically quantify the trade-off between improved payment fit for some groups and reduced payment fit for others.

### A summary measure of selection incentives

As noted previously, to quantify the trade-off emerging with constrained regressions we need to identify the groups of interest and develop a method for comparing the under- and overcompensation for these groups.

#### Which groups are relevant in terms of risk selection actions?

Newhouse (1993) defines risk selection as “actions by consumers and health plans to exploit unpriced risk heterogeneity and break pooling arrangements. Often the term selection is also used to refer to the outcome of these actions”. In other words: risk selection is about actions (by health plans and consumers) with the intention and/or the outcome that undercompensated *groups* are (to some extent) separated from overcompensated *groups*. This implies that evaluation of a RE model starts with stipulating the groups that can be targets of risk selection actions. How would this work? For example, if plans can *only* take actions that discriminate between people under the age of 65 and those above the age of 65, these become the groups of concern when it comes to (measuring) risk selection (incentives). A RE formula fully addresses selection incentives in this circumstance if it eliminates incentives to favor one group over the other. Analogously, if plans can only discriminate on the basis of “yes/no chronic condition” then these are the two relevant groups. If health plans can discriminate on combinations of “yes/no > 65” and “yes/no chronic condition”, there will be four groups of concern, and so on.

Some research defines groups according to a single geographic indicator under the thinking that a health plan might favor or disfavor certain regions because of systematic differences in medical spending, as was done in a study of Germany by Bauhoff [2]. Other research defines groups according to the services used, the idea being that a health plan could favor or disfavor primary versus some kinds of specialty care, for example, to encourage/discourage potential enrollees anticipating making use of those services.^13^ Since the instruments for health plans to engage in risk selection differ across health care schemes, there is no universal set of relevant groups. Thus, the first step for evaluating incentives for risk selection in a particular setting is to identify the possible selection actions in that setting and to derive the relevant groups. For example, in the Netherlands health plans are unable to discriminate at the individual level due to open enrollment requirements. On the other hand, however, plans can discriminate across groups on the basis of network design. For example, contracting with first-best physicians for treatment of disease X will attract patients with disease X; conversely, a poor network in terms of quality or convenience will deter patients in that disease group.

#### What is the relative importance of under- and overcompensations for these groups?

Once the relevant groups in terms of selection actions have been identified, the second step for evaluating RE models is to compare the importance of the under- and overcompensations for these groups. Literature on selection incentives provides at least four arguments as to why the relative importance of under- or overcompensations may differ across groups and vary with the size of these under- and overcompensations. A first argument comes from van Barneveld et al. [26] who contend that small predictable profits and losses are likely to be irrelevant for a health plan. Selection can be costly and the net benefits are uncertain, and small incentives may simply not induce a health plan to act. A second argument derived from standard welfare economics is made by Layton et al. [16] who show that the welfare loss from price distortions due to under- or overcompensation by a RE model is proportional to the square of the payment gap, implying that the inefficiency from selection goes up more than proportionally with the magnitude of the under- and overcompensation. A third argument can be drawn from the work by Ellis and McGuire [9] who argue that selection incentives do not just depend on an indicator’s predictiveness (how well the indicator co-varies with total health care spending) but also on predictability of that indicator (how well the indicator can be anticipated) and demand responsiveness of individuals scoring on that indicator. For example, Ellis and McGuire find that both “use of durable medical equipment” and “use of anesthesia” are indicators with high predictiveness but that the first indicator is much more predictable (and therefore much more vulnerable to service level distortion) than the latter. This point implies that potential selection inefficiency of under- or overcompensation for one group can be larger than that of an equal under- or overcompensation for other groups. A fourth argument comes from van de Ven et al. [29] who contend that the degree of inefficiency depends on the specific selection actions that can occur as a consequence of under- or overcompensation. They distinguish many selection actions, such as selective advertising, offering a choice of deductibles, making supplementary insurance (un)attractive for certain groups, offering group contracts, and quality skimping. They argue that of all possible selection actions “quality skimping” is a special threat to the functioning of regulated health plan markets because it not only reduces market efficiency, but *also* the quality of medical care. Incentives for quality skimping, however, are only present when groups with relatively strong preferences for high quality are *under*compensated.^14^ After all, if these groups would be overcompensated, health plans would have incentives to *improve* quality of care. Thus, for groups with strong preferences for high quality — presumably those with a chronic condition — undercompensation may be worse than overcompensation.^15^

#### A summary measure of under- and overcompensation for relevant groups

To formalize our ideas about a summary measure for quantifying the trade-off between better-targeted compensation for some groups and worse-targeted compensation for others, consider a set of selection actions that allows health plans to discriminate among G mutually exclusive groups indexed by *g* with *g* = 1, …, G.^16^ We can then use data to determine:

- s_g_ the share of the population in group g, with ∑_g_s_g_ = 1,
- $\bar{r}_{\text{g}}$ the average plan revenue for a person in group g,
- $\bar{c}_{\text{g}}$ the average plan cost for a person in group g, and
- $\bar{r}_{\text{g}} - \bar{c}_{\text{g}}$, the average under/overcompensation for a person in group g.

Given these parameters, under- and overcompensations can be summarized by $\mathop \sum \nolimits_{\text{g}} {\text{s}}_{\text{g}} \left| {\bar{r}_{\text{g}} - \bar{c}_{\text{g}} } \right|$, i.e., the sum of absolute under- and overcompensations weighted by the share of the affected population. We follow standard assumptions (used in calculation of both predictive ratios and over/undercompensation) by regarding *medical* spending as plan cost and figuring over- and undercompensation for an *average* plan.^17^ With this, in the Dutch context, over- and undercompensation is solely a function of the RE payments.^18^ Specifically, average plan revenues equals average predicted spending from the RE model and $\left| {\bar{r}_{\text{g}} - \bar{c}_{\text{g}} } \right|$ boils down to absolute residual spending for group g from the RE model. Moreover, $\mathop \sum \nolimits_{\text{g}} {\text{s}}_{\text{g}} \left( {\bar{r}_{\text{g}} - \bar{c}_{\text{g}} } \right)$ equals zero.

As a next step, we incorporate the relative importance of under- and overcompensation by weighting these for each group. As discussed above, the literature provides arguments for different types of weighting. Since our empirical application is intended as “a proof of concept,” we will simply apply one type of weighting, which is raising under- and overcompensation to a power *p*. This weighting function is common in the statistical and economic literature on plan payment: individual and group *R*-squared measures are obviously based on squared deviations^19^ and in welfare economics, the efficiency loss associated with a price distortion (such as a tax) is proportional to the square of the distortion. In our empirical analyses, we apply several variants of this weighting function with powers ranging from 1 to 2.^20^ We thus propose a summary measure of the form:

1 $${\mathcal{L}}\mathop { = \sum }\limits_{\text{g}} s_{\text{g}} \left| {\bar{r}_{\text{g}} - \bar{c}_{\text{g}} } \right|^{p} \quad {\text{with }}1 \le p \le 2.$$

The summary measure ${\mathcal{L}}$ is intuitive, weighting under/overcompensation raised to a power by the share of the affected population, and is easy to compute. ${\mathcal{L}}$ has a minimum value of 0 and no upper bound. Comparing ${\mathcal{L}}$ for RE models is meaningful only for comparing models estimated on the same data with the same definitions of group membership. In our empirical analyses we estimate all RE models on exactly the same data with exactly the same group definition. Under this procedure, RE scheme 1 will be said to be preferred to scheme 2 if ${\mathcal{L}}_{\text{1}} < {\mathcal{L}}_{2}$.

As we noted at the outset of this section the relevant form of Eq. (1) depends on stipulation of the relevant groups and definition of the relative importance of under- and overcompensation, which are not straightforward decisions. Taking these factors into account would ideally be based on an elicitation of the concerns of regulators and an analysis of what actions plans can take and the welfare consequences of these actions. We intend the current paper to be a “proof of concept” of the idea of using constraints in a RE model. We select a subset of the groups that are relevant in terms of selection and assume a simple weighting function of under- and overcompensation.

As we explain in the “Data and empirical methods” Section, our groups are categorized by a set of indicators included in the current Dutch RE model and a set of indicators omitted from the model. The utility of constraints on regression coefficients emerges when at least one indicator is omitted from the RE model. We will use the included and the omitted indicators jointly to define mutually exclusive groups for the entire Dutch population, and compute ${\mathcal{L}}$ for this partition of the population. We also will compute ${\mathcal{L}}$ for the two sets of included and omitted indicators separately. These two partial classifications allow us to show the effect of constraints on groups identified by the included versus the omitted indicators. Tightening the constraint improves things for the omitted indicators but imposes a cost on fit among the included indicators.

## Data and empirical methods

### Data

The empirical analyses are based on administrative data including individual-level information on medical spending and risk indicators for almost the entire Dutch population in 2012 (*N* = 16.5 million). These data come from various sources, including health plans, tax authorities and the registration service for social benefits.^21^ The resulting merged data are those used to estimate the RE model for health plan payment in the Netherlands in 2015. As a first step in our analyses we faithfully replicate this model, such that our “base model” accurately indicates expected over- and undercompensation in the Netherlands for 2015 [8]. Our alternative RE models and simulations modify this base model and are estimated on the same data. Here we briefly describe the risk indicators included in the base model and provide some general statistics.

The Dutch RE model for 2015 is the product of more than 20 years of research and experience and includes the following indicators: 40 risk classes based on an interaction between age and gender, 25 risk classes based on the use of specified prescription drugs in the previous year referred to as pharmacy-based cost groups or PCGs [15], 16 risk classes based on diagnostic information from hospital treatment in the previous year referred to as diagnoses-based cost groups or DCGs [23, 33], seven risk classes for people with high costs in multiple prior years referred to as multiple-year high cost groups or MHCGs [32], five risk classes based on the use of durable medical equipment in the previous year referred to as durable medical equipment cost groups or DMECGs [31], four risk classes based on an interaction between two age groups and yes/no “PCG + DCG + MHCG + DMECG > 0”, 12 risk classes based on an interaction between socioeconomic status and age, 10 risk classes based on regional characteristics and 19 risk classes based on an interaction between source of income and age. All risk indicators have been carefully developed in research programs sponsored by the Dutch Ministry of Health. For further details on these risk indicators, see van Kleef et al. [30].^22^

The RE model of 2015 was estimated by a least-squares regression with medical spending in 2012 as the dependent variable and the risk classes described above as 138 independent dummy variables.^23^ Medical spending includes the expenses on primary care, pharmaceuticals, hospital inpatient and outpatient care, maternity care, obstetrics and medical devices among other categories, but excludes expenses on mental health care and home health nursing care.^24^ Although the latter two categories of spending are included in the mandatory benefit package of 2015, they are omitted from the main RE model, with funds allocated for them using a separate RE model. In this paper we will be concerned with the primary RE model used to allocate more than 80% of health care costs among health plans in the Netherlands in 2015 [8]. We refer to the RE model of 2015 as the “base model”.

Table 1 provides some information on the prevalence of risk characteristics and the distribution of medical spending in our data. For simplicity of presentation we report aggregated risk categories instead of all 138 explanatory variables separately. Average spending in the population equals 1848 euros per person per year. Not surprisingly, average spending is relatively high for people age 65 years or older, those who receive a disability benefit, people living at an address with more than 15 residents (which approximates being in an institution for long-term care, which is paid for via a separate insurance scheme) and those in a PCG, DCG, DMECG and/or MHCG. The latter four are the most direct indicators of morbidity; nearly 23% of the population is classified by at least one of these indicators.

**Table 1:** Population frequency and medical spending (in euros, 2012) at aggregated levels of risk characteristics (*N* = 16.5 million)

	Population frequency (%)	Medical spending	
		Mean	SD
Men, <65	42	1207	5893
Men, ≥65	8	4612	11,050
Women, <65	41	1487	5212
Women, ≥65	9	4123	8889
Region, clusters 1–5	50	1979	6941
Region, clusters 6–10	50	1719	6237
Source of income, reference group (age <18 or >64)	38	2477	8235
Source of income, disability benefits	5	3817	10,570
Source of income, social security benefits	2	2321	7110
Source of income, student	3	588	2717
Source of income, self-employment	4	1012	3814
Source of income, other (including employment)	48	1282	4541
Socioeconomic status, home address >15 residents	1	4507	10,219
Socioeconomic status, income deciles 1–3	30	1842	6526
Socioeconomic status, income deciles 4–7	40	1869	6527
Socioeconomic status, income deciles 8–10	30	1721	6555
Pharmacy-based cost group (PCG)			
No	82	1212	5199
Yes	18	4751	10,417
Diagnoses-based cost group (DCG)			
No	91	1353	4921
Yes	9	6855	14,530
Durable medical equipment cost group (DMECG)			
No	99	1772	6382
Yes	1	10,933	17,099
Multiple-year high cost group (MHCG)			
No	94	1378	4957
Yes	6	9536	17,056
PCG, DCG, DMECG and/or MHCG			
No	77	984	4106
Yes	23	4784	11,090
Total population	100	1848	6597

In addition to the administrative data, we use health survey information to assess how constraints on undercompensation for one omitted group affect estimates of over- and undercompensation for a series of other omitted groups of interest. The survey was conducted in 2011 among a representative sample of the Dutch population and includes a broad range of questions on general health status, physical impairments, mental health problems, particular chronic diseases and prior utilization of medical care. A unique, anonymous person identifier allows merging the survey information with the administrative data. We calculate under- and overcompensation for survey groups as the predicted expenses from a RE model estimated on the administrative data (*N* = 16.5 million) minus the actual expenses. In contrast to the administrative data, the survey data are available only for a small sample (*N* = 14,310) of the population, implying that under- or overcompensation for groups identified from the survey may be vulnerable to random variation. We report on groups for which under- or overcompensation by the base model is statistically significant. For the specific definition of these groups see van Veen et al. [35], Appendix 2).

### Selecting study indicators included and study indicators omitted from the RE model

For our empirical application we assume that health plans are able to discriminate on the basis of the following information: “yes/no use of home care in the previous year”, “yes/no use of physiotherapy in the previous year”, and DCGs. The first two indicators are omitted from the Dutch RE model while the DCGs are included. This set of indicators allows study of how constrained regression methods affect fit for groups defined by both omitted and included indicators.

The Dutch DCGs are a hierarchical categorization of persons based on selected diagnostic information from inpatient or outpatient hospital treatment in the previous year.^25^ Persons are classified in a DCG if they received at least one of these selected treatments in the previous year. The diagnostic cost group (DCG) categorization partitions the population into 16 mutually exclusive groups. As shown in Table 2, the “No DCG” group, those with none of the selected treatments in the previous period, account for 91% of the population. Where the “No DCG” group has below average medical expenses, the higher DCGs have above average expenses. Since all DCGs are explicitly included as dummy regressors in the Dutch RE model, average predicted spending for these groups perfectly fits average actual spending (see footnote 4), implying that for all DCGs the average under/overcompensation is zero (see Table 2).

**Table 2:** Population frequency, medical spending and under/overcompensation by the Dutch RE model of 2015 (base model) in euros (2012) for the 4 omitted and 16 included indicators studied in our empirical analyses (*N* = 16.5 million)

	Population frequency (%)	Medical spending		Under/overcompensation base model
		Mean	SD	
Omitted indicators				
Use of home care in *t*−1				
No	97.31	1659	5985	34
Yes	2.69	8696	16,541	−1231
Use of physiotherapy in *t*−1				
No	97.62	1737	6313	23
Yes	2.38	6422	13,124	−922
Included indicators				
No DCG	91.00	1353	4921	0
DCG1	0.67	5573	8943	0
DCG2	1.49	4649	8100	0
DCG3	1.11	4196	8243	0
DCG4	1.80	5058	9541	0
DCG5	1.16	6291	11,420	0
DCG6	1.26	7645	13,461	0
DCG7	0.55	8832	15,511	0
DCG8	0.12	10,039	15,978	0
DCG9	0.30	9582	18,583	0
DCG10	0.33	13,175	20,678	0
DCG11	0.04	14,557	25,078	0
DCG12	0.07	17,107	28,243	0
DCG13	0.04	25,105	41,154	0
DCG14	0.04	90,296	42,858	0
DCG15	0.01	62,451	110,800	0
Total population	100	1848	6597	0

Each of our omitted indicators “yes/no use of home care in the year *t*−1” and “yes/no use of physiotherapy in the year *t*−1” partitions the population into two mutually exclusive groups.^26^ Given the zero-sum principle of the Dutch RE model (and the constrained models estimated in our empirical analyses), reductions in undercompensation for the “yes” groups imply corresponding reductions in overcompensation for the complementary “no” groups. For example, if the undercompensation for users of home care reduces by 40%, the overcompensation for the complementary group of non-users will reduce by 40% too. For simplicity of presentation we primarily focus on the two “yes” groups. As shown in Table 2, these two groups comprise 2.7% and 2.4% of the population in year *t*, respectively. Both groups are systematically undercompensated by the current Dutch RE model. So far, however, the Dutch government has not found *appropriate* risk adjustor variables to indicate membership in these particular groups [37]. For some analyses we convert the two yes/no indicators into four mutually exclusive categories by crossing the indicators and classifying the population as having none, home care only, physiotherapy only, or both indicators in the previous year.

### Constraining coefficients in the RE models

We introduce a series of constraints to the base RE model that limit the under/overcompensation for one or more omitted indicators with a fixed percentage. This works as follows.

For any person i, the RE payment is *Y*_i_ = ∑_j_b_j_x_ij_ where *x*_ij_ is the value of the included 0/1 indicator j for person i, and b_j_ is the weight on the indicator in the RE formula. If the number of people in the group of interest g is n_g_ the average payment for a member of group g is:

2 $$\bar{Y}_{\text{g}} = \frac{1}{{n_{\text{g}} }}\mathop \sum \limits_{{{\text{i}} \in {\text{g}}}} \mathop \sum \limits_{\text{j}} b_{\text{j}} x_{\text{ij}} .$$

This can be rewritten as:

3 $$\bar{Y}_{\text{g}} = \mathop \sum \limits_{\text{j}} b_{\text{j}} \bar{x}_{\text{gj}} ,$$

where $\bar{x}_{\text{gj}}$ is the mean value of indicator variable j for group g. This group mean must be calculated on an initial pass through the data. The constraints then take the form of setting $\bar{Y}_{\text{g}}$ equal to a target value which can be easily implemented with the RESTRICT statement in the PROC REG procedure in SAS. This constraint is simply an equation linear in the coefficients of the RE model, resulting in coefficient estimates that maximize the fit of the model as measured by an *R*-squared given that the compensation for g equals the specified value. The target value for $\bar{Y}_{\text{g}}$ can be chosen as any amount; here, we reduce undercompensation for the omitted group(s) to a fixed portion α of the undercompensation by the base model. This can be written as:

4 $$\bar{Y}_{\text{g}} = \bar{c}_{\text{g}} - \alpha (\bar{c}_{\text{g}} - \mathop \sum \limits_{\text{j}} b_{\text{j,OLS}} \bar{x}_{\text{gj}} ),$$

where $\bar{c}_{\text{g}}$ equals the average plan costs for a person in group g and $\mathop \sum \nolimits_{\text{j}} {\text{b}}_{{{\text{j}},{\text{OLS}}}} \bar{x}_{\text{gj}}$ equals the average plan revenue under the base model (OLS) for a person in group g.

An interesting feature of constraints in this form is that they do not require that information on yes/no membership of g is available for the *entire* risk pool. Instead, it is sufficient to have a good approximation of the average per person medical spending for g, as well as the mean indicators values conditional on g [see Eq. ((3)]. Information on these parameters obtained from a decent *sample* of the risk pool (e.g., respondents of a health survey) could be sufficient.

### Empirical analyses

As shown in Table 2, the Dutch RE model of 2015 (our “base model”) leads to an average per person undercompensation of 1231 euros for users of home care in the previous year and 922 euros for users of physiotherapy in the previous year. Given these magnitudes, we begin by estimating a series of constrained regressions, where in each case there is just one constraint. For each of the two omitted indicators we reduce the undercompensation in series by 20, 40, 60, 80 and 100%. For each model we calculate measures of overall fit (*R*-squared and Cummings prediction measure (CPM)) as well as measures for group fit (under- or overcompensation for included and omitted groups).^27^ To quantify the trade-off between improved fit for omitted groups and deteriorated fit for included groups we track a series of our summary measure (1) based on the mutually exclusive set of four groups identified by the omitted indicators, the mutually exclusive set of the 16 included DCGs, and for the cross-product of the two sets (64 groups). We also consider a range of powers to apply to the payment gap for each group of interest in order to check the sensitivity of results to the form of the weighting function. In a next step, guided by the results for the single constraints, we try several combinations of constraints for the two omitted indicators to check whether two constraints can produce better overall model performance than any single constraint. We find a superior two-constraint specification that performs better than any model with a single constraint.

## Results

Table 3 shows results for the base RE model and for the same model supplemented with a series of single constraints to reduce undercompensation for the group with use of home care in year *t*−1. Since the constraint will bind, the constrained models will always yield a lower *R*-squared than the unconstrained base RE model. The incremental loss in *R*-squared goes up as the constraint is more binding, but the absolute magnitude of the reduction in *R*-squared is always very small: the most binding constraint in which the undercompensation for users of home care in year *t*−1 is completely eliminated decreases the *R*-squared by only 0.3 percentage points. Thus, in terms of the *R*-squared, the costs of the constraint appear to be very low. In terms of the CPM (not bound to fall in a “least-squares” regression) the constrained model even leads to better fit than the base model, though the actual improvement is relatively minor.

**Table 3:** Results (euros, 2012) for the base model and for ten single-constraint models

	*R*-squared (×100%)	CPM (×100%)
Base model	22.5%	24.8%
Base model + single constraint to limit undercompensation for users of *home care* in *t*−1 by:		
20%	22.5%	24.9%
40%	22.5%	24.9%
60%	22.4%	24.9%
80%	22.3%	24.9%
100%	22.2%	24.8%
Base model + single constraint to limit undercompensation for users of *physiotherapy* in *t*−1 by:		
20%	22.5%	24.9%
40%	22.5%	25.0%
60%	22.4%	25.0%
80%	22.2%	25.0%
100%	22.0%	24.8%

**Fig. 1:**
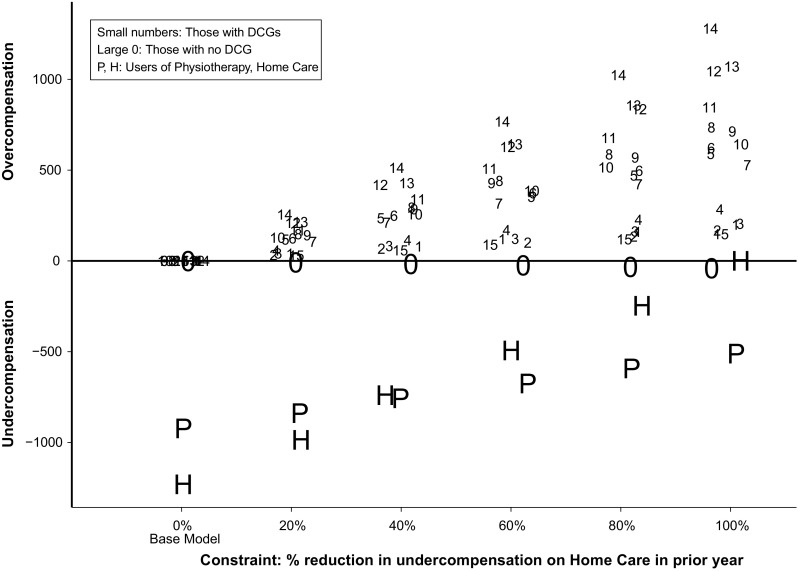
Results (euros, 2012) for the base model (*N* = 16.5 m) and for the same model supplemented with a single constraint to reduce undercompensation in year *t* for users of home care in *t*−1

To assess group-level fit, Fig. 1 presents results for the sets of included and omitted groups. The capital letters H and P represent the undercompensation in year *t* for users of home care and physiotherapy in year *t*−1, respectively. By design, the undercompensation for users of home care in year *t*−1 is smaller for the constrained models than for the base model. More interesting is the reduction of undercompensation for users of physiotherapy in year *t*−1, showing that reducing undercompensation for one omitted group can also improve compensation for another omitted group. Apparently, certain risk indicators in the RE model are positively correlated with both the home care group and the physiotherapy group. When weights on these risk indicators are altered by the constraint, undercompensation for the physiotherapy group is reduced as well.

Figure 1 also shows that, as expected, the constraint for the omitted group introduces over- and undercompensation for the group indicators included in the RE model. The small numbers represent the overcompensation for the 15 DCGs while the large “0” represents the undercompensation for those without a DCG. For the latter group the constrained models introduce an undercompensation up to 40 euros per person per year; for the DCGs the models with constraints introduce overcompensation up to 1280 euros per person per year. The direction of these under- and overcompensations can be explained by the positive correlation between the omitted groups and the DCGs: since the home care and physiotherapy groups have relatively high proportions of people in a DCG (not shown here), the constrained model overcompensates the DCGs in order to move funds to these omitted groups.^28^ Like the reduction in undercompensation for users of home care, the change in under- or overcompensation for the other groups in Fig. 1 is also linear, a consequence of constrained least-squares estimators with linear constraints.^29^

**Fig. 2:**
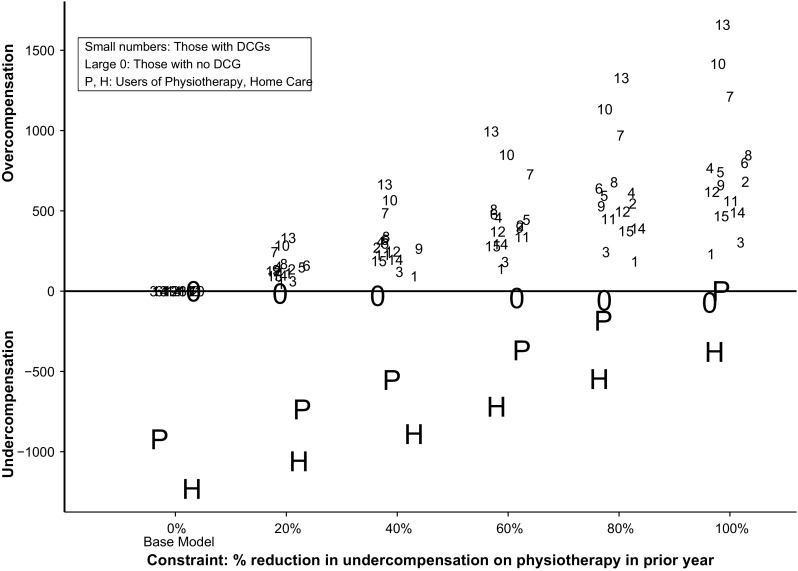
Results (euros, 2012) for the base model (*N* = 16.5 m) and for the same model supplemented with a single constraint to reduce undercompensation in year *t* for users of physiotherapy in *t*−1

Figure 2 reports the analogous results for the base model supplemented with a series of single constraints reducing the undercompensation for users of physiotherapy in the previous year. Patterns are similar to those in Fig. 1, with the difference that the constraint regarding physiotherapy leads to bigger changes in under- or overcompensation for other groups (both the included groups and the other omitted group).

**Fig. 3:**
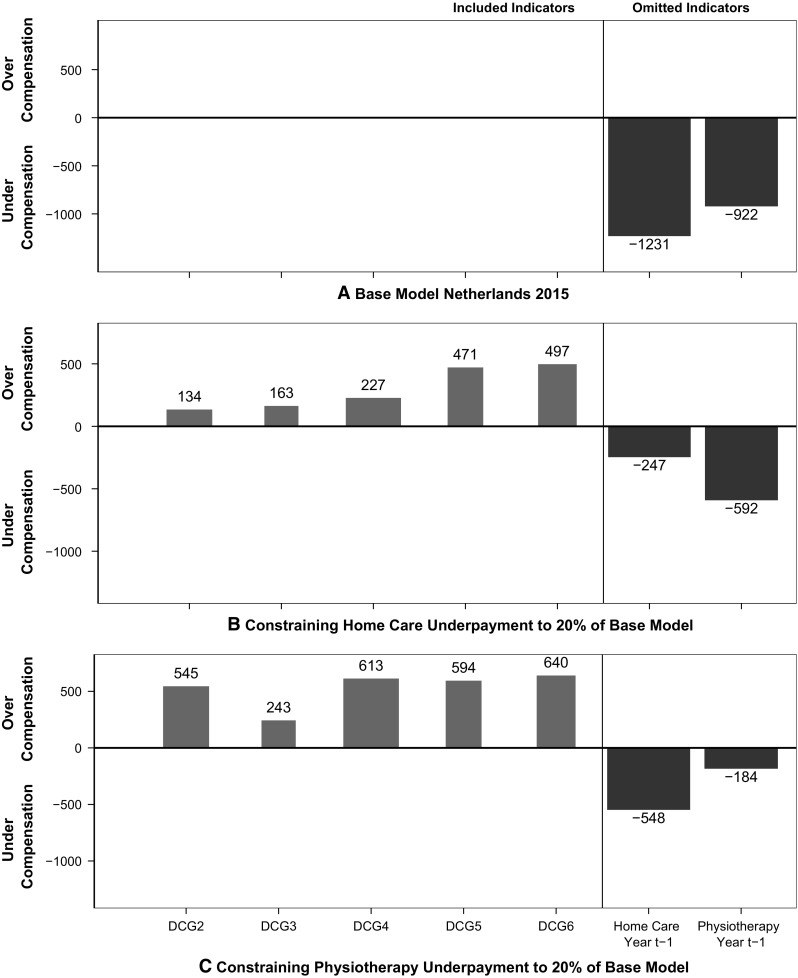
Over- and undercompensation in year *t* in three models (*N* = 16.5 m)

Introduction of a constraint involves a trade-off between a reduction of undercompensation for omitted groups and an increase in under- or overcompensation for the included ones. When it comes to incentives for risk selection, however, it is not only the under- or overcompensation that matters, but also the size of the affected group. Figure 3 combines these two aspects, showing selected results for three of the RE models from Figs. 1 and 2. The height of the bars indicates the average under- or overcompensation for a group and the width indicates the relative size of the group. The product of height and width, as represented by the area of the bars, indicates the total under- or overcompensation for a group. The right side of panel A shows the undercompensation for the two omitted indicators in the base model. The left side of the panels tracks the overcompensation for the larger of the included groups (DCGs with at least 1% of the population included). With no constraint, least-squares estimators eliminate over- or undercompensation in the base model for the included groups. Panel B shows results for the restriction of reducing the undercompensation for the home care group by 80% compared to the base model. Undercompensation for both omitted groups falls, as was reported in Fig. 1, and overcompensation appears for the DCGs shown in the Figure. Panel C shows the same set of results for one of the models in Fig. 2. Overall, Fig. 3 illustrates that constraining undercompensation for one omitted group pushes funds towards that specific group as well as to the other omitted group, and to “sick people” in general, at least as indicated by a DCG. For the DCGs in Fig. 3 this appears as an overcompensation for members of these groups.

Though the group results in Figs. 1, 2 and 3 are informative, they cannot evaluate trade-offs between the improvement for omitted groups and the worsening for included groups. As argued in Section “Theory and concepts” of this paper, evaluating the trade-off can be based on a mutually exclusive grouping of individuals and a weighting of under- and overcompensation, as is done by our summary measure proposed in Section “Theory and concepts”. The essence of the summary measure in (1) is that weighted under- or overcompensations are computed and aggregated for mutually exclusive groups.

**Fig. 4:**
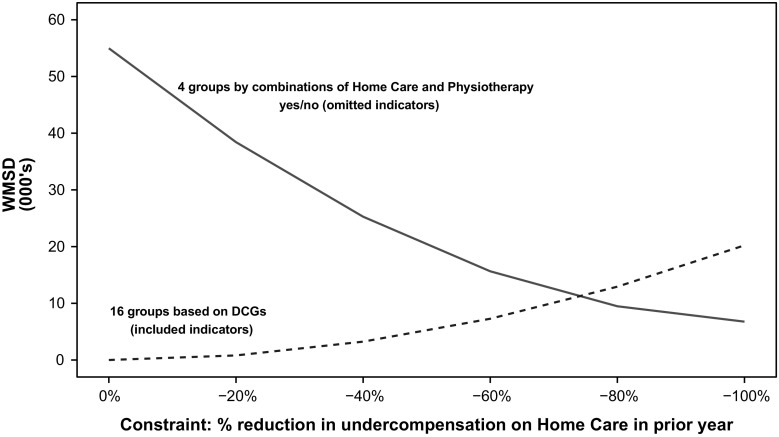
Base model (*N* = 16.5 m) with constraints on undercompensation for users of home care in prior year: weighted mean squared deviation (WMSD) for two sets of mutually exclusive groups

Figure 4 illustrates application of our summary measure to the base model and to the same model with a single constraint for reducing the undercompensation for the home care group. The measure is computed according to formula (1) with *p* = 2, separately for two sets of groups: the four combinations of yes/no home care use and yes/no physiotherapy use in the previous year (solid line) and the 16 DCG-groups (dotted line). In the case of *p* = 2, we refer to our measure as the weighted mean squared deviation (WMSD). The results clearly show the trade-off between the improvement for the omitted groups and the deterioration for included groups.

**Fig. 5:**
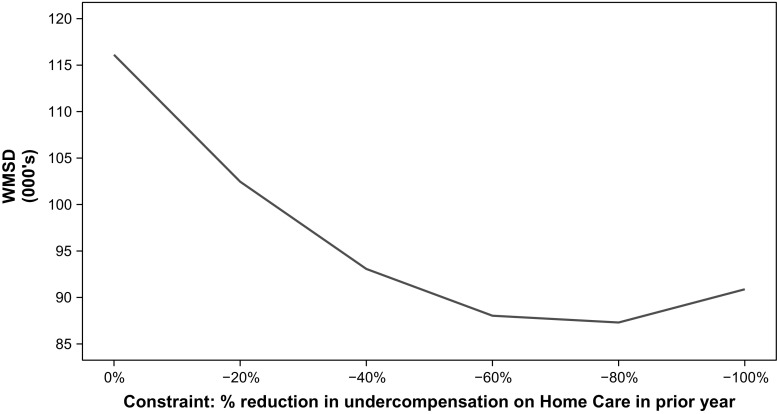
Base model (*N* = 16.5 m) with constraints on undercompensation for users of home care in prior year: weighted mean squared deviation (WMSD) for full population segmented into 64 groups by home care (Y/N), physiotherapy (Y/N) and 16 DCGs

Whereas Fig. 4 illustrates application of the measure separately for omitted and included groups, Fig. 5 integrates the selection incentives on omitted and included groups in a single measure. As in Fig. 4, the measure is calculated according to formula (1) with *p* = 2, but this time for all 64 mutually exclusive combinations of the four omitted and 16 included groups. Up to an 80% reduction in undercompensation for home care, the constraint reduces the WMSD, but further tightening the restriction for the omitted group increases the WMSD because of deterioration in the fit of compensation for the included groups. Based on these results we conclude that for mutually exclusive combinations of the selected included and omitted groups studied in this paper, a well-chosen constrained model can outperform the base model.

**Fig. 6:**
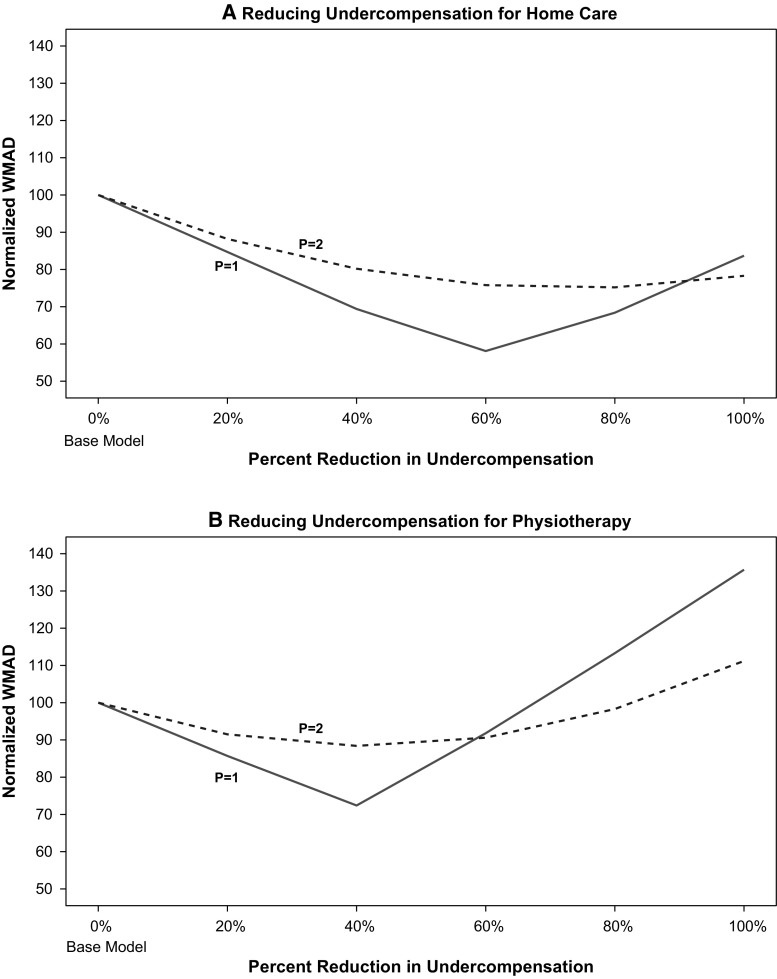
Normalized weighted mean absolute deviations (euros, 2012) for 64 mutually exclusive groups for exponential weights on deviations of *p* = 1 and *p* = 2 (*N* = 16.5 m)

To check sensitivity of our results to different assumptions about the weighting of under- and overcompensations, we calculated the summary measure (as presented in Fig. 5) for values of *p* ranging from 1.0 to 2.0. Figure 6 displays the normalized values of weighted mean absolute deviations (WMAD, a more general term to describe our measure for values of *p* other than 2.0) for the end points of 1.0 and 2.0. These are normalized so that the WMAD for each set of model comparisons is set at 100 for the base model. The pattern is similar for *p* = 1 and *p* = 2 (also for the intermediate values of *p* not shown). In panel A, the measure falls as undercompensation is reduced for users of home care, but after some point in the 60–80% reduction range, it goes up. The findings for reducing undercompensation for users of physiotherapy shown in panel B are similar. For both weights of the over- and undercompensation, although the exact minimum varies slightly, the same U-shape describes the results. Thus, our finding that a moderate reduction in undercompensation minimizes our measure is insensitive to reasonable weights for the absolute value of the over- and undercompensation.

The results presented above clearly show that for a mutually exclusive set of the selected included and omitted indicators, a single-constraint model can outperform the base model. A natural next question is whether adding a second constraint to the model can lead to further improvements. To check this in our case we started with the most effective single constraint for home care according to our summary measure with *p* = 2: reducing the undercompensation for home care by 80%. (This is the minimum of the U-shaped dotted line corresponding to *p* = 2 from Fig. 6a). We introduced the additional constraint that the undercompensation for physiotherapy should be reduced by 20% from the base model, and then should be reduced by 40%. The value of the summary measure (the WMSD) fell by slightly more than 1% of its value with the first constraint at a target undercompensation for physiotherapy of 20% less, but then went up as the second constraint was tightened to the 40% drop in undercompensation. While the improvement obtained by introducing the second constraint is considerably less when the first constraint is roughly optimized, the results show that in terms of the summary measure applied here, a two-constraint model can outperform a single-constraint model.

**Fig. 7:**
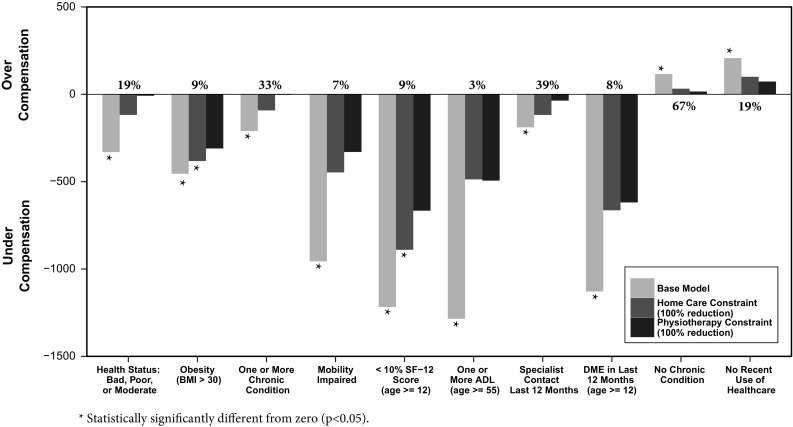
Population frequency (%) and average under- or overcompensation (euros, 2012) for prior-year survey-based indicators not included in the base model and not included in constraints (*N* = 14,310)

Compared to OLS, constrained regressions inherently increase under/overcompensation for groups explicitly recognized in the RE model. At the same time, they can reduce under/overcompensation for groups omitted from the RE model *but* explicitly recognized in the constraints. The effect of constrained regression on other omitted groups, however, is less obvious. To gain insight in this effect we examined the consequences of constraints for modifying undercompensation for home care and physiotherapy users on a series of groups identified by health survey information. As described in the data section, the small sample size (*N* = 14,310) implies that under- or overcompensations for groups can be vulnerable to random variation. Figure 7 shows results for the ten groups for which the initial under- or overcompensation by the base model is statistically significant (*p* ≤ 0.05).^30^ The results are striking: a single constraint for reducing the undercompensation for users of home care (or for users of physiotherapy) in the previous year can also substantially reduce under- or overcompensation for other omitted groups. Apparently, certain risk indicators in the RE model are correlated with both the home care (physiotherapy) group and the groups presented in Fig. 7. When weights on these risk indicators are altered by the constraint, this reduces under- or overcompensation for the groups in Fig. 7 as well.

For example, consider the group on the left-hand side of Fig. 7, those reporting their health status to be in the lowest three categories: bad, poor or moderate, composing 19% of the population. These people are undercompensated by an average of 331 euros in the base model. If we impose the constraint eliminating the undercompensation for home care, the undercompensation for the bad-poor-moderates falls to 118 euros, and if we impose instead the constraint that we eliminate the undercompensation for physiotherapy, the undercompensation disappears altogether (to only 8 euros). Remarkably, for all eight of the undercompensated groups, imposing either constraint has a meaningful favorable impact on the undercompensation. The constraints also improve payments for the groups that were overcompensated, as shown on the right-hand side of Fig. 7. The 67% of the population with no chronic illness were overcompensated by 116 euros in the base model, and this is cut to 32 with the home care constraint imposed and 16 if the physiotherapy constraint is imposed.

The findings in Fig. 7 have two important implications. First, the observation that under- and overcompensations for the groups in the figure change substantially as a result of a constraint implies that an appropriate trade-off between the benefits and costs of a constraint requires involving *all* groups of interest. This emphasizes the importance of the questions raised in Sect. “Theory and concepts”, i.e., “Which groups are relevant in terms of risk selection actions?” and “What is the relative importance of under- and overcompensation for these groups?” Second, the direction of the changes in under- and overcompensation in Fig. 7 implies that the benefits of constrained regression may reach far: a *single* constraint intended to improve payment fit for one relevant omitted group can lead to an improvement for many others.

## Discussion

The natural way to improve RE models for particular groups is to add new/better risk adjustor variables indicating membership in these groups. But what if appropriate risk adjustor variables are not (yet) available? For these situations, this paper proposed constraining the estimation coefficients of the RE model in order to reduce the under- or overcompensation of the group(s) of interest to a fixed amount. Compared to OLS, constraints reduce model fit in terms of *R*-squared. Our empirical application of constrained regression to the Dutch RE model of 2015, however, shows that the magnitude of this reduction may be small. An alternative fit measure, the Cummings prediction measure (CPM) also changes little with the introduction of the constraints considered here. On the basis of our results, an *R*-squared or CPM should be supplemented with other measures when evaluating constrained regression models, since *R*-squared and CPM appear to be insensitive to changes in group-level fit induced by the introduction of the constraints considered here.

At a group level, a constraint limiting under- or overcompensation for an omitted group comes at the cost of introducing under- or overcompensation for included groups. To illustrate our proposed method, we have chosen potentially relevant groups to study and selected a general summary measure to quantify the trade-offs involved. With these assumptions, we find that the improvement for omitted groups can outweigh the deterioration for included groups. Moreover, multiple constraints can reduce selection incentives over a single constraint. Although we study a particular application of constrained regressions, we have no reason to think these findings are special to this empirical setting. If an indicator for an omitted group of interest is correlated with variables already included in the RE model, it should generally be possible to introduce at least a modest constraint that makes first-order cuts in undercompensation for the group with the omitted indicator at the cost of only “marginal” over/undercompensation for groups based on included indicators. It will be worthwhile to investigate the conditions (if they exist) under which introduction of a constraint at the margin is associated with a model improvement, perhaps using envelope-theorem type arguments. In any case, the practical performance of constraints in a particular plan payment application is straightforward to assess systematically for each setting.

This paper is intended to be a “proof of concept.” Ultimately, to be useful in terms of plan payment redesign, application of constrained regression methods requires stipulating the groups that can be a target of selection actions and valuing the under- or overcompensation for these groups. This exercise starts with identifying the possible selection-related actions in a certain context. For example, when insurers might discriminate on the basis of “yes/no use of home care in the prior year”, these are the two relevant groups to distinguish. When insurers are able to discriminate on the basis of combinations of “yes/no use of home care in the prior year” and “yes/no inclusion in any DCG”, these are the four relevant groups to distinguish, and so on.^31^ A mismatch between the (potential) selection targets and the groups distinguished in the summary measure may result in misleading outcomes. So, compared to standard measures used for evaluation of risk equalization models — such as the *R*-squared, CPM and predictive ratios — our summary measure is more complex since it requires information about (potential) selection actions in a certain context (as a basis for stipulating the relevant groups) and the effects of these actions (as a basis for valuing the under- and overcompensations for these groups). When it comes to selection incentives, however, our measure is also more meaningful.

We believe our approach offers an opportunity to expand the role of regulators and public policy makers. Rather than being *reactive* to problems identified in empirical studies of RE models, regulators can be *proactive* and take steps to define the objectives that will be maximized by RE model estimation. Further research is clearly necessary to find a process by which a social consensus can be reached about defining groups of concern for protection against incentives for selection. Once consensus has been reached, however, the “objective” of a RE model can be quantified in the form of a measure like the one used in this paper. Optimization of an explicit objective function with respect to payment weights on included indicators implies a new way for estimating parameters of a RE model. We regard this to be an important area for ongoing research on selection incentives and risk equalization.

## Footnotes

1 By “health plan competition” we mean competition among health insurers who offer one or multiple health plans. A “health plan” refers to a health insurance product. All consumers who have the same “health plan” have an identical contract with the same insurer concerning benefits coverage, cost-sharing, quality, services, etc. Since objectives and strategies of insurers can differ across health plans, this paper will speak of health plans instead of insurers as decision makers.

2 Newhouse [20] defines risk selection as actions by consumers and health plans to exploit unpriced risk heterogeneity and break pooling arrangements. Often the term selection is also used to refer to the outcome of these actions.

3 By “health plan payment scheme” we mean the total set of payment features, which can include risk equalization, reinsurance, risk corridors and premiums among other features.

4 The expected value of an OLS residual conditional on a dummy variable regressor is zero. The statistical residual from an OLS model is the individual-level over- or underpayment in a RE model based on the regression coefficients.

5 In the presence of such risk adjusters, providing more home care and physiotherapy in the current year will lead to higher RE payments in the next year.

6 A closely related and well-known application of this approach can be found in the Appendix “On Optimal Insurance Policies” of Kenneth Arrow’s *Uncertainty and the welfare economics of medical care* [1].

7 The form of constraints we use is written out in the “Data and empirical methods” Section of this paper. See, in particular, Eq. (2).

8 An example of an *R*-variable might be an indicator for smoking. Smoking may predict higher medical spending but it may be undesirable to “reward” the health plan of a smoker by higher RE payments.

9 The Rothschild-Stiglitz model was adapted to managed health care insurance by Glazer and McGuire [12]. See Breyer et al. [3] for a recent review.

10 Cao and McGuire [4] in Medicare and Eggleston and Bir [6] in employer-based insurance find patterns of spending on various services consistent with service-level selection among competing at-risk plans. Ellis et al. [7] rank services according to incentives to undersupply them. Consistent with service-level selection, they show that HMO-type plans tend to underspend on services (in relation to the average) just as the selection index predicts. This pattern of spending is not observed among enrollees in unmanaged plans. See also Carey [5].

11 Defining subgroups of the population on the basis of predicted spending can lead to predictive ratios close to 1.0 even if the prediction model itself is weak, and therefore is not necessarily a mark in favor of the RE model. Unless the model itself does a good job at differentiating high- from low-cost individuals, a predictive ratio according to a ranking by predicted spending is not very informative.

12 Other measures of individual and group fit have been proposed and applied in the literature (see [36] for a review).

13 See Ellis and McGuire [9] for implementation of this approach in Medicare and McGuire et al. [19] for its application in marketplaces.

14 In a zero-sum RE payment scheme, undercompensation of the chronically ill implies overcompensation of the complementary group of healthy individuals, and vice versa.

15 Note that constrained regression can be a tool for changing undercompensation for groups of chronically ill into overcompensation. A related argument is made by Lorenz [17] who also identifies empirical methods that weight over- and undercompensation asymmetrically.

16 The discussion in this section draws on Layton et al. [16] who derive a similar summary measure starting with conditions for profit maximization by a health plan.

17 This avoids concerns with selection across plans, differential premiums, and differential plan efficiency.

18 In practice multiple payment features may coexist (e.g., risk equalization and reinsurance). Practical use of our summary measure should incorporate all these relevant features. For example, reinsurance figures into plan payments in marketplaces in the US. Simulating payments recognizing these plan payment features can be used in calculating predictive ratios or over/undercompensation [16, 32].

19 See Van Veen et al. [36] for discussion of the various statistical measures applied to evaluation of RE schemes.

20 Layton et al. [16] propose a metric similar to (1) with p = 2, squaring the payment-cost gap for each group, also appealing to the usual form of welfare loss in economics, in which the area of a welfare triangle is proportional to the square of a discrepancy between the first-best and the actual price. Their selection metric measures improvement in incentives over a payment system with no premium categories and no RE, and falls between 0 and 1. Lorenz [16] considers situations in which over- and undercompensation may impose different magnitudes of losses, and estimators that would be appropriate for minimizing the asymmetric loss functions.

21 In estimating the RE model for 2015, medical spending from 2012 has been adjusted to reflect mandatory coverage changes in the period 2013–2015. In a second stage coefficients were linearly adjusted for expected cost inflation. For reasons of simplicity we excluded the second stage from our analysis.

22 A complete description of the Dutch payment system would include some subsidiary (and less well-developed) RE models for some small cost categories, and description of some of the regional adjustments built into the main model. These details are not important for our simulations. Readers are referred to Eijkenaar et al. [8] for full details on the RE models.

23 Analogous to the RE methodology in the Netherlands, medical spending in 2012 (dependent variable) is annualized by dividing actual individual-level costs of 2012 by the fraction of the year an individual was enrolled in the basic health insurance in 2012. Subsequently, this “fraction” is included as a weight in the estimation model and computation of means. For example, an individual who was enrolled for 6 months and had 1000 euros expenses is given annualized costs of 2000 euros (1000/0.5) and a weight of 0.5.

24 Since medical expenses for home care itself are excluded, the undercompensation of about 1200 euros on users of home care implies that prior use of home care has predictive value for other types of medical expenses. A possible explanation is that users of home care have a relatively high probability of dying.

25 The DCG classification was developed in several steps. First, a team of medical experts carefully determined whether or not diagnoses refer to a chronic condition. Diagnoses referring to a chronic condition were categorized in 144 more or less clinically homogeneous groups, which — in a next step — were clustered into 15 DCGs based on residual cost (according to a prediction model including explanatory variables based on age, gender and PCGs) using Ward’s hierarchical clustering method. If enrollees have multiple diagnoses that would fall into different DCGs, they are classified in only one DCG, i.e., the one with the highest estimated coefficient. For further details about the Dutch DCGs see Van Kleef et al. [33].

26 These indicators are based on the use of home care and physiotherapy as far as covered by the Dutch basic benefit package of 2015. This benefit package fully covers the use of home care. Physiotherapy is only covered for treatment of certain chronic conditions and above a threshold of 20 visits.

27
*R*-squared is computed as $1 - \frac{{\mathop \sum \nolimits_{{{\text{i}} = 1}}^{\text{n}} ({\text{Y}}_{\text{i}} - \hat{Y}_{\text{i}} )^{2} }}{{\mathop \sum \nolimits_{{{\text{i}} = 1}}^{\text{n}} ({\text{Y}}_{\text{i}} - \bar{Y})^{2} }}$. CPM is computed as $1 - \frac{{\mathop \sum \nolimits_{{{\text{i}} = 1}}^{\text{n}} |{\text{Y}}_{\text{i}} - \hat{Y}_{\text{i}} |}}{{\mathop \sum \nolimits_{{{\text{i}} = 1}}^{\text{n}} |{\text{Y}}_{\text{i}} - \bar{Y}|}}$.

28 Payment weights patterns under a constrained regression are consistent with the finding in Glazer and McGuire [12] that optimal risk adjusted weights should reflect the correlation between indicators part of the risk equalization and omitted factors affecting health care expenses.

29 This can be shown by writing out the formula for the estimated coefficients in constrained least squares. Suppose we seek to estimate a vector *β* of regression coefficients on *j* variables *X* subject to *q* linear constraints of the form *Q* ^T^ where *Q* is a j × q matrix of full rank and the superscript *T* indicates the transpose of a matrix. *Y* is a q × 1 vector of constants. One of these q equations could be interpreted in our context as a constraint that the average payment for a target group is equal to an amount “*y*.” The constrained estimator is $\hat{\beta }^{c} = \hat{\beta } - \left( {X^{\text{T}} X} \right)^{ - 1} Q\left( {Q^{\text{T}} \left( {X^{\text{T}} X} \right)^{ - 1} Q} \right)^{ - 1} (Q^{\text{T}} \hat{\beta } - \, y)$. From this it is clear that ${\text{d}}\hat{\beta }^{\text{c}} /{\text{dy}}$ is constant.

30 The test is a two-sided *t*-test that the difference between payment and cost for individuals in each group equals zero.

31 This can be illustrated with the outcomes of the model that constrains the estimated coefficients such that the average undercompensation for people with use of home care in the prior year equals zero. While for the entire group of people with use of home care in the prior year the average undercompensation indeed equals zero (see Fig. 1), this is not the case for subsets of this group on the basis of “yes/no inclusion in any DCG”: those without a DCG (1.7% of the population) are undercompensated by 41 euros while those with a DCG (1.0% of the population) are overcompensated by 65 euros (not shown in Tables and Figures). This implies that evaluation of incentives for selection under a certain risk equalization model requires knowledge about the level at which selection (potentially) takes place.
